# CB_1_ receptor activation induces intracellular Ca^2+^ mobilization and 2-arachidonoylglycerol release in rodent spinal cord astrocytes

**DOI:** 10.1038/s41598-018-28763-6

**Published:** 2018-07-12

**Authors:** Zoltán Hegyi, Tamás Oláh, Áron Kőszeghy, Fabiana Piscitelli, Krisztina Holló, Balázs Pál, László Csernoch, Vincenzo Di Marzo, Miklós Antal

**Affiliations:** 1 Department of Anatomy, Histology and Embryology, Faculty of Medicine, University of Debrecen, 4032 Debrecen, Hungary; 2 Department of Physiology, Faculty of Medicine, University of Debrecen, 4032 Debrecen, Hungary; 3 Endocannabinoid Research Group, Institute of Biomolecular Chemistry, Consiglio Nazionale delle Ricerche, 80078 Pozzuoli, Naples Italy; 4 MTA-DE Neuroscience Research Group, University of Debrecen, 4032 Debrecen, Hungary; 5 Present Address: Department of Cognitive Neurobiology, Center for Brain Research, Medical University of Vienna, 1090 Vienna, Austria

## Abstract

Accumulating evidence supports the role of astrocytes in endocannabinoid mediated modulation of neural activity. It has been reported that some astrocytes express the cannabinoid type 1 receptor (CB_1_-R), the activation of which is leading to Ca^2+^ mobilization from internal stores and a consecutive release of glutamate. It has also been documented that astrocytes have the potential to produce the endocannabinoid 2-arachidonoylglycerol, one of the best known CB_1_-R agonist. However, no relationship between CB_1_-R activation and 2-arachidonoylglycerol production has ever been demonstrated. Here we show that rat spinal astrocytes co-express CB_1_-Rs and the 2-arachidonoylglycerol synthesizing enzyme, diacylglycerol lipase-alpha in close vicinity to each other. We also demonstrate that activation of CB_1_-Rs induces a substantial elevation of intracellular Ca^2+^ concentration in astrocytes. Finally, we provide evidence that the evoked Ca^2+^ transients lead to the production of 2-arachidonoylglycerol in cultured astrocytes. The results provide evidence for a novel cannabinoid induced endocannabinoid release mechanism in astrocytes which broadens the bidirectional signaling repertoire between astrocytes and neurons.

## Introduction

Astrocytes were long thought to play only a supporting role in the central nervous system. However, the discovery that Ca^2+^ transients in astrocytes are coupled to the enhancement or depression of neuronal activity has led to the recognition that astrocytes may play a substantial role in neural information processing^1–3^. Since then, many details of a bidirectional communication between astrocytes and neurons has been demonstrated^4–6^. Astrocytes express various neurotransmitter receptors, such as glutamatergic and purinergic receptors^7^, the activation of which leads to the mobilization of Ca^2+^ from intracellular stores^8,9^. In turn, astrocytes release neuroactive substances called gliotransmitters, like glutamate, D-serine and ATP^10^, which modulate neuronal excitability and synaptic transmission^11–14^.

Endocannabinoids are also implicated in this bidirectional signaling^15–17^. It is well established that in addition to neurons, astrocytes also express cannabinoid type-1 receptors (CB_1_-R) both in the brain^16^ and spinal cord^18,19^. Navarrate and Araque showed that in the hippocampus endocannabinoids released by neurons activate CB_1_-Rs on astrocytes, which - in contrast to activation of CB_1_-Rs on neurons - leads to phospholipase C (PLC)-dependent Ca^2+^ mobilization from internal stores and a consecutive release of glutamate that activates NMDA receptors in adjacent pyramidal neurons^16,17^.

It has also been shown that astrocytes have the potential to produce one of the best-known endogenous CB_1_-R agonist 2-arachidonoylglycerol (2-AG)^20–22^. The synthesis of 2-AG in astrocytes can be evoked by ATP or endothelin^20,22,23^, requires a sustained increase in the intracellular calcium concentration ([Ca^2+^]_i_) and the subsequent activation of the PLC-diacylglycerol lipase alpha (DGLα, the synthesizing enzyme for 2-AG) pathway^22–24^.

Although astrocytes express CB_1_-Rs and DGLα^19,25,26^, the spatial co-expression of these proteins in astrocytes has never been investigated. In addition, though activation of CB_1_-Rs on astrocytes leads to Ca^2+^ mobilization^16,17^ and Ca^2+^ transients may evoke the production of 2-AG^22–24^, 2-AG release from astrocytes evoked by CB_1_-R activation has never been demonstrated. Filling up these gaps in our knowledge seems particularly important given that endocannabinoid mediated neuron-astrocyte-neuron signaling appears to be crucial in many functions of the central nervous system including pain processing in the spinal dorsal horn. Therefore, in the present study, we investigated whether spinal astrocytes can release 2-AG in response to activation of their CB_1_-Rs.

## Results

### Astrocytes in the superficial spinal dorsal horn express CB_1_-Rs and DGLα in close proximity

Almost half and one third of the astrocytic profiles show positive immunostaining for CB_1_-R and DGLα, respectively, in the superficial spinal dorsal horn^19,26^. Extending the scope of the previous studies, first we investigated whether astrocytes that carry CB_1_-Rs also express DGLα.

**Figure 1:**
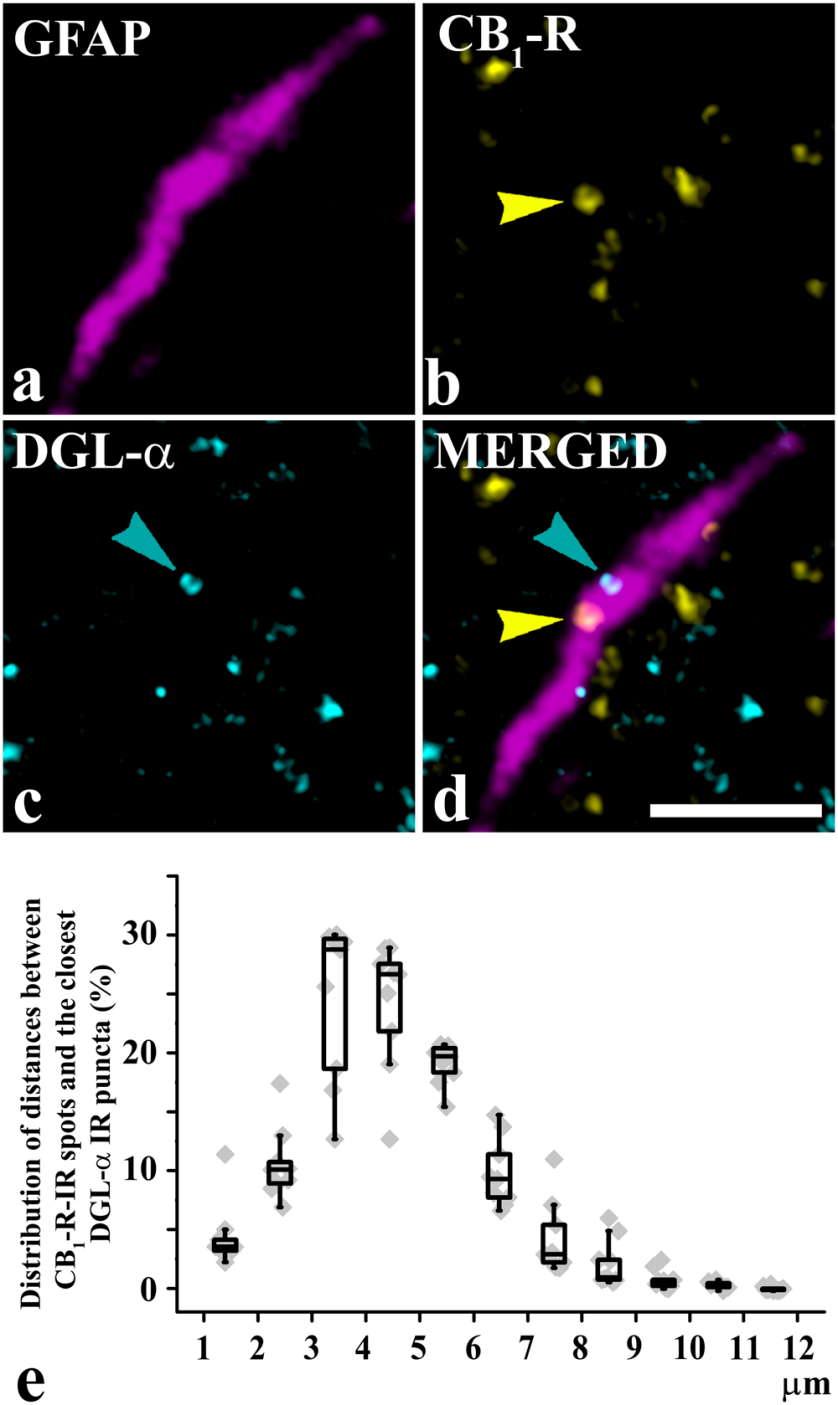
Astrocytes express CB_1_-Rs and DGLα in close proximity to each other in the rat superficial spinal dorsal horn. (**a–d**) Micrographs of a single 1-µm-thick laser scanning confocal optical section illustrating the co-localization between immunolabeling for GFAP (a marker for astrocytes, magenta; (**a**) CB_1_-R (yellow; **b**) and DGLα (cyan; **c**). Two puncta immunoreactive for CB_1_-R or DGLα that are located close together within the confines of a profile stained for GFAP are marked with arrows and appear in mixed colors in the merged image (**d**). Scale bar: 5 µm. (**e**) Box plot showing the distribution of distances between CB_1_-R immunoreactive spots and the closest DGLα immunoreactive spots that were recovered within the confines of GFAP immunoreactive profiles.

First, we obtained sections of lamina I-II of the rat spinal dorsal horn and carried out triple immunostainings for glial fibrillary acidic protein (GFAP; a marker for astrocytes), CB_1_-Rs and DGLα. We observed strong immunolabeling for all three molecules. Investigating 155 GFAP-immunoreactive (GFAP-IR) profiles, we found that 46.3 ± 2.5% of GFAP-IR profiles displayed CB_1_-IR puncta and 37.5 ± 2.0% displayed DGLα-IR puncta. Most interestingly, 75.3 ± 2.7% of CB_1_-R positive GFAP-IR profiles displayed immunostaining also for DGLα, whereas 91.7 ± 1.3% of DGLα positive GFAP-IR profiles showed immunostaining also for CB_1_-R. This indicates a high degree of co-localization between CB_1_-R and DGLα. Moreover, CB_1_-R-IR and DGLα-IR spots were quite close together (Fig. 1a–d) - the closest DGLα-IR spot was never located more than 12 µm from a CB_1_-R-IR spots. DGLα-IR spots showed a unimodal distribution around CB_1_-R-IR spots, with a peak at 4 µm (Fig. 1e).

### Activation of CB_1_-Rs intensifies slow intracellular Ca^2+^ transients in astrocyte-like cells in the superficial spinal dorsal horn

The activation of CB_1_-Rs on hippocampal astrocytes leads to PLC-dependent mobilization of Ca^2+^ from cytoplasmic stores^15–17,24^, resulting in an increase in [Ca^2+^]_i_ in a 10–20 µm long segment of astrocytic processes^27^. Because an increase in [Ca^2+^]_i_ can lead to the stimulation of DGLα and the production of 2-AG^20,22,28^, the close co-localization of CB_1_-R and DGLα raises the possibility that spinal astrocytes might produce 2-AG following CB_1_-R activation.

**Figure 2:**
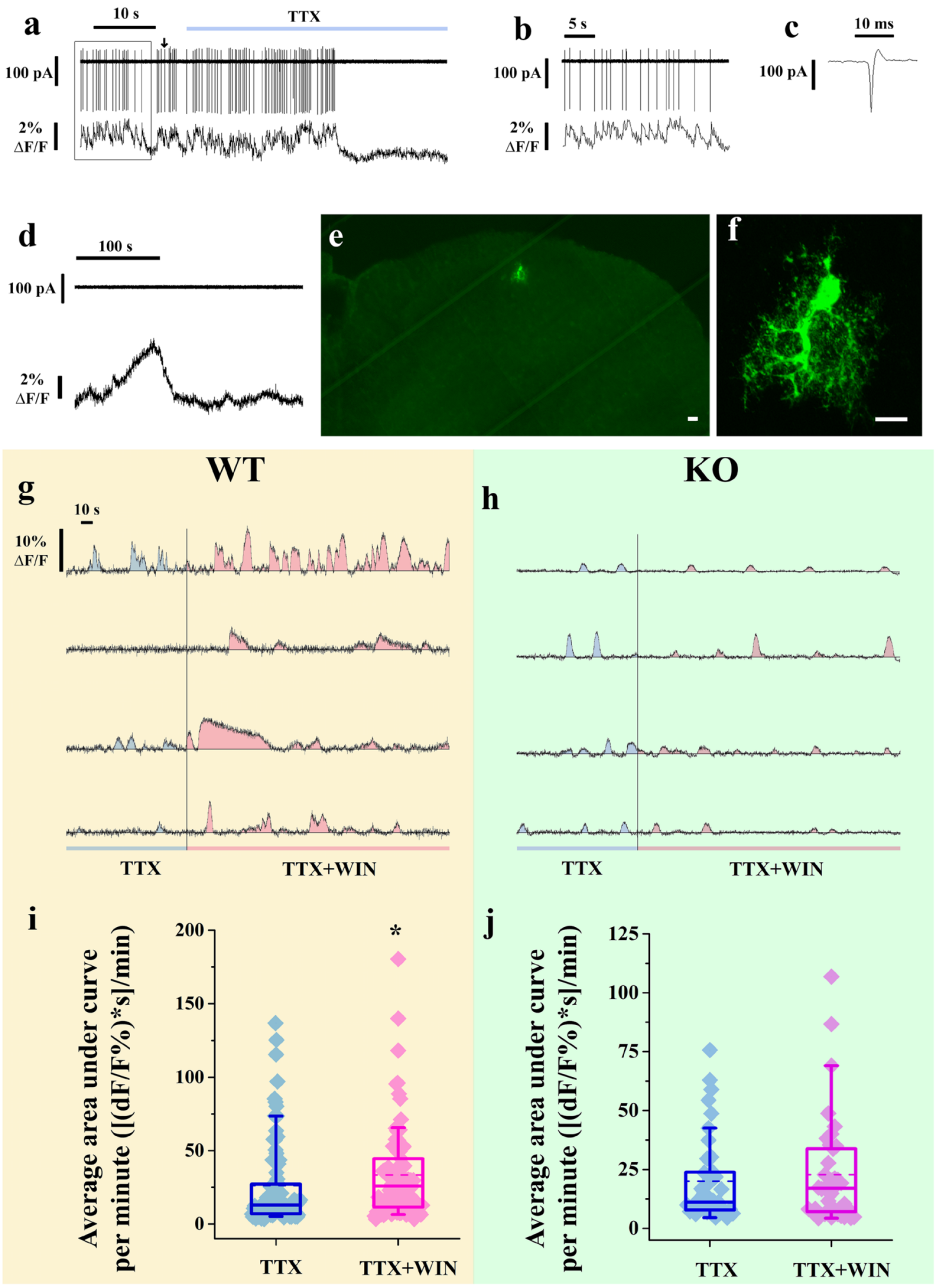
Application of CB_1_-R agonist amplifies intracellular calcium transients of astrocytes within the superficial spinal dorsal horn. (**a–c**) Records obtained with simultaneous loose patch recording (upper trace) and calcium imaging (lower trace) of a single cell. Application of 1 µM TTX (blue bar) diminishes both action potentials and calcium transients. The segment of the recording outlined in **a** is shown in **b** with an expanded time scale. The single event indicated by an arrow in **a** is shown expanded in **c**. (**d**) Records obtained with simultaneous loose patch recording (upper trace) and calcium imaging (lower trace) of a single cell. Slow calcium transients are not coupled to action potentials. (**e**,**f**) Confocal images of at low (**e**) and high (**f**) magnifications of a cell labeled intracellularly with biocytin from which action potential-uncoupled slow calcium transients were recorded. Scale bars: 20 µm (**e**) and 10 µm (**f**). (**g,h**) Six minutes long records illustrating changes in [Ca^2+^]_i_ of cells showing slow calcium transients in wild type (**g**) and CB_1_-R knock out (**h**) mice, during treatment with 1 µM TTX (first 2 minutes), and 1 µM TTX + 1 µM WIN (last 4 minutes). The application of WIN increases both the frequency and amplitude of spontaneous slow calcium transients in the wild type (**g**) but not in the CB_1_-R knock out (**h**) animals. (**i,j**) Box-plots of average areas under the curves of intracellular calcium transients recorded from cells showing slow calcium transients in wild type (**i**) and CB_1_-R knock out (**j**) mice during TTX and TTX + WIN application.

As a first step in investigating this possibility, we tested whether activation of CB_1_-Rs on spinal astrocytes evokes an increase in [Ca^2+^]_i_. To distinguish between neurons and astrocytes, we observed both electrophysiological current activities and the dynamics of calcium events in the recorded cells. Slices cut from the lumbar spinal cord of 10- to 15-day-old mice were loaded with the calcium indicator Oregon Green BAPTA-1-AM. Spontaneous electrical activity was recorded simultaneously with calcium imaging in individual cells in the superficial spinal dorsal horn, using extracellular loose patch recording. Activity in the recorded cells showed two different types of kinetics. Some cells presented rapid, short-duration calcium events, always preceded by a fast current spike in simultaneous loose-patch recordings (Fig. 2a–c). After blocking voltage gated sodium channels with 1 µM tetrodotoxin (TTX), the calcium events as well as the current spikes vanished (Fig. 2a), indicating that the recorded cells were neurons. Other cells presented slow calcium transients that were not coupled with current spikes (Fig. 2d). Since slow intracellular calcium transients in other areas of the central nervous system are characteristic to glial cells^29^, we predicted that these cells were astrocytes. To test this, we filled some of the cells intracellularly with biocytin. The morphology of the labeled cells was characteristic of astrocytes (Fig. 2e,f), confirming our prediction.

Next, we investigated the effect of the CB_1_-R agonist WIN 55,212-2 (WIN) on the [Ca^2+^]_i_ of cells that showed spontaneous slow calcium transients (assumed to be astrocytes). To block action potential firing and associated neurotransmitter release in the spinal cord slices, thus minimizing possible indirect effects evoked by the activation of neuronal CB_1_-Rs, we supplemented the extracellular solution with 1 µM TTX. After recording spontaneous calcium events from individual cells in this condition, we applied 1 µM WIN in the presence of TTX. The application of WIN substantially increased both the frequency and amplitude of the calcium transients (Fig. 2g). To compare the calcium events recorded before and after the application of WIN, we calculated the area under the curve (AUC) for the entire control period and the entire drug application period for 73 cells recorded in slices obtained from three mice. Then we determined the average AUC per minute of recording and found that the average AUC during the control period was 29.0 ± 3.8, which increased significantly to 36.1 ± 4.1 (p = 0.0029) after the application of WIN (Fig. 2i).

To verify that the enhanced calcium events represented by the increase in AUC values were the result of CB_1_-R activation, we carried out negative control experiments on slices cut from the lumbar spinal cord of 10–15-day-old CB_1_-R knock out mice. Under identical experimental conditions as those used for recordings from wild-type animals, the application of WIN did not change the frequency and amplitude of the calcium transients in the absence of CB_1_-Rs (Fig. 2h). The AUC values calculated for 40 cells recorded in slices obtained from two knock out mice were 20.0 ± 2.9 before and 22.7 ± 3.6 after the application of WIN (p = 0.8725; Fig. 2j).

The results clearly demonstrate that the activation of CB_1_-Rs leads to an increase in [Ca^2+^]_i_ in astrocytes within the superficial spinal dorsal horn.

### Spinal astrocytes express CB_1_-Rs and DGLα also in culture

**Figure 3:**
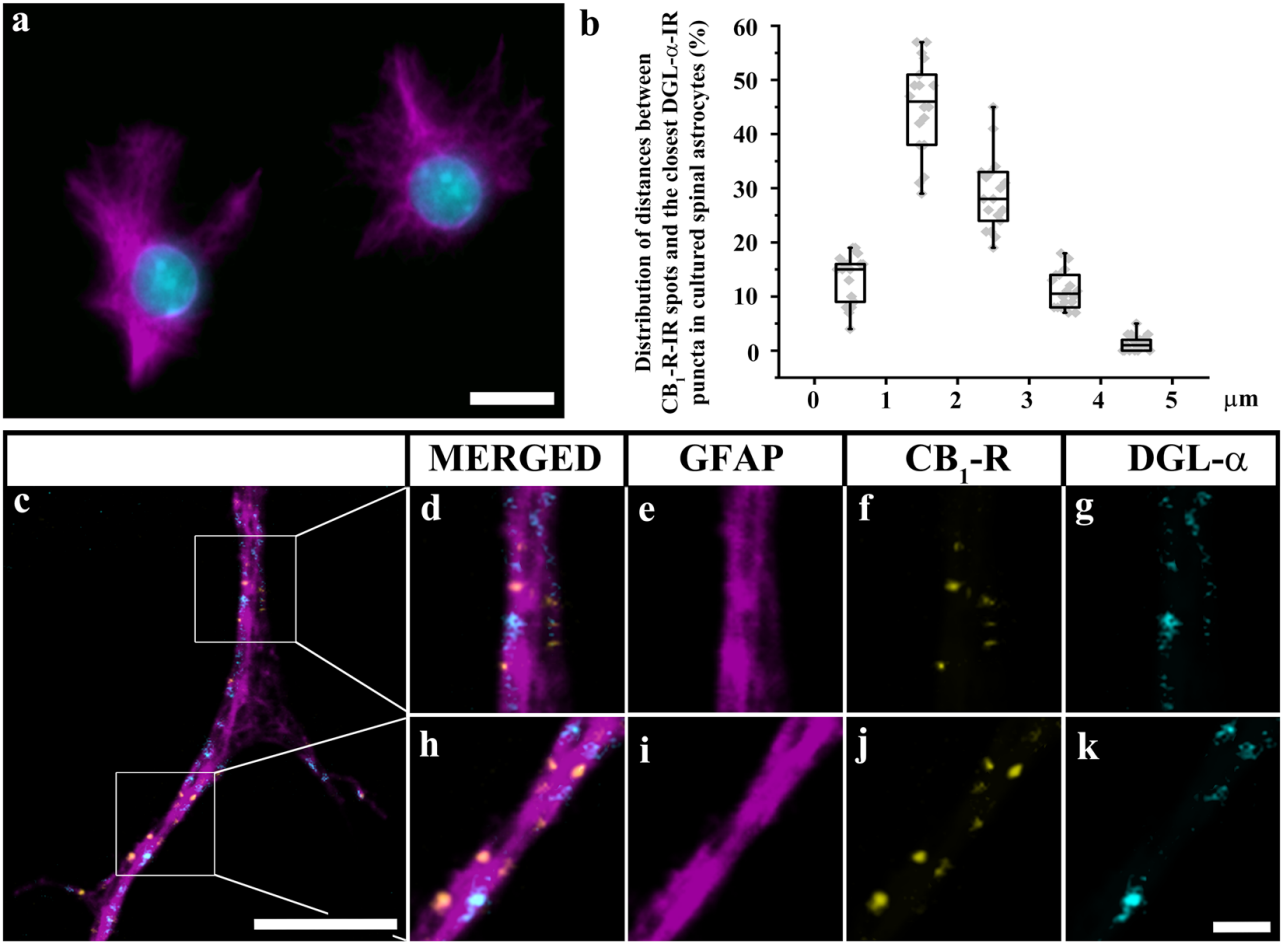
Cultured spinal astrocytes express CB_1_-Rs, and DGLα in close proximity to each other. (**a**) Micrograph of a single 1 µm thick laser scanning confocal optical section illustrating cells immunostained with GFAP (magenta) in primary cell culture of spinal astrocytes. (The DAPI stained cell nuclei appear in cyan). (**b**) Box-plot histogram showing the distribution of distances between CB_1_-R immunoreactive spots and the closest DGLα immunoreactive spots that were recovered within the confines of GFAP immunoreactive cultured astrocytes. (**c–k**) Micrographs of a single 1 µm thick laser scanning confocal optical section illustrating the co-localization between immunolabeling for GFAP (a marker for astrocytes, magenta; **c,e,i**), CB_1_-R (yellow; **c,f,j**) and DGLα (cyan; **c,g,k**) in a process of a cultured spinal astrocyte. (**d–k**) Enlarged segments of a GFAP immunoreactive astrocytic process (**c**) are illustrated. Puncta immunoreactive for CB_1_-R and DGLα are located within the confines of the glial process stained for GFAP. They appear in mixed colors in the merged images (**c,d,h**). Note that CB_1_-R and DGLα immunoreactive spots are located close to each other within the confines of GFAP immunolabelled cultured astrocyte. Scale bars: 10 µm (**a**), 5 µm (**c**), 1 µm (**d–k**).

Next, we intended to test whether the CB_1_-R-evoked increase in [Ca^2+^]_i_ has any effect on astroglial 2-AG mobilization. However, measuring 2-AG released by astrocytes in the central nervous system is far from trivial. Therefore, we generated primary cultures of spinal astrocytes from 7–9 day-old rats and mice (Fig. 3a), and conducted the rest of the experiment in a cell culture environment.

To show that cultured astrocytes express CB_1_-R and DGLα similarly to spinal astrocytes *in vivo*, we carried out triple immunostaining for GFAP, CB_1_-Rs and DGLα, and evaluated the immunolabeling similarly to the *in situ* conditions. We investigated 180 and 167 GFAP-IR astrocytes in cell cultures cultivated from rat and wild type mouse spinal cord, respectively.

In the rat cultures, 29.9 ± 21.9% of GFAP-IR cells displayed CB_1_-IR puncta and 28.9 ± 1.8% displayed DGLα-IR puncta. The overlap between CB_1_–R and DGLα immunostaining was remarkably high; 81.2 ± 2.2% of CB_1_-R positive GFAP-IR cells displayed immunostaining also for DGLα, whereas 84.1 ± 1.7% of DGLα positive GFAP-IR cells showed immunostaining also for CB_1_-R. CB_1_-R-IR and DGLα-IR spots were very close to each other (Fig. 3c–k)- the closest DGLα-IR spot was never located more than 5 µm from a CB_1_-R-IR spots. DGLα-IR spots showed a unimodal distribution around CB_1_-R-IR spots, with a peak between 1–2 µm (Fig. 3b).

Results obtained from the wild type mouse cultures were practically identical to those collected from rat cultures in terms of co-localization and distribution pattern. In this case, 29.4 ± 1.3% and 29.3 ± 2.0% of GFAP-IR cells displayed CB_1_-R-IR and DGLα-IR, respectively; and 86.4 ± 1.2% of CB_1_-R positive GFAP-IR cells displayed immunostaining also for DGLα, whereas 86.7 ± 2.1% of DGLα positive GFAP-IR cells showed immunostaining also for CB_1_-R. The distances between the CB_1_-R-IR puncta and the closest DGLα-IR spots showed here also a unimodal distribution with a peak at 2 µm and a maximum value of 5 µm.

**Figure 4:**
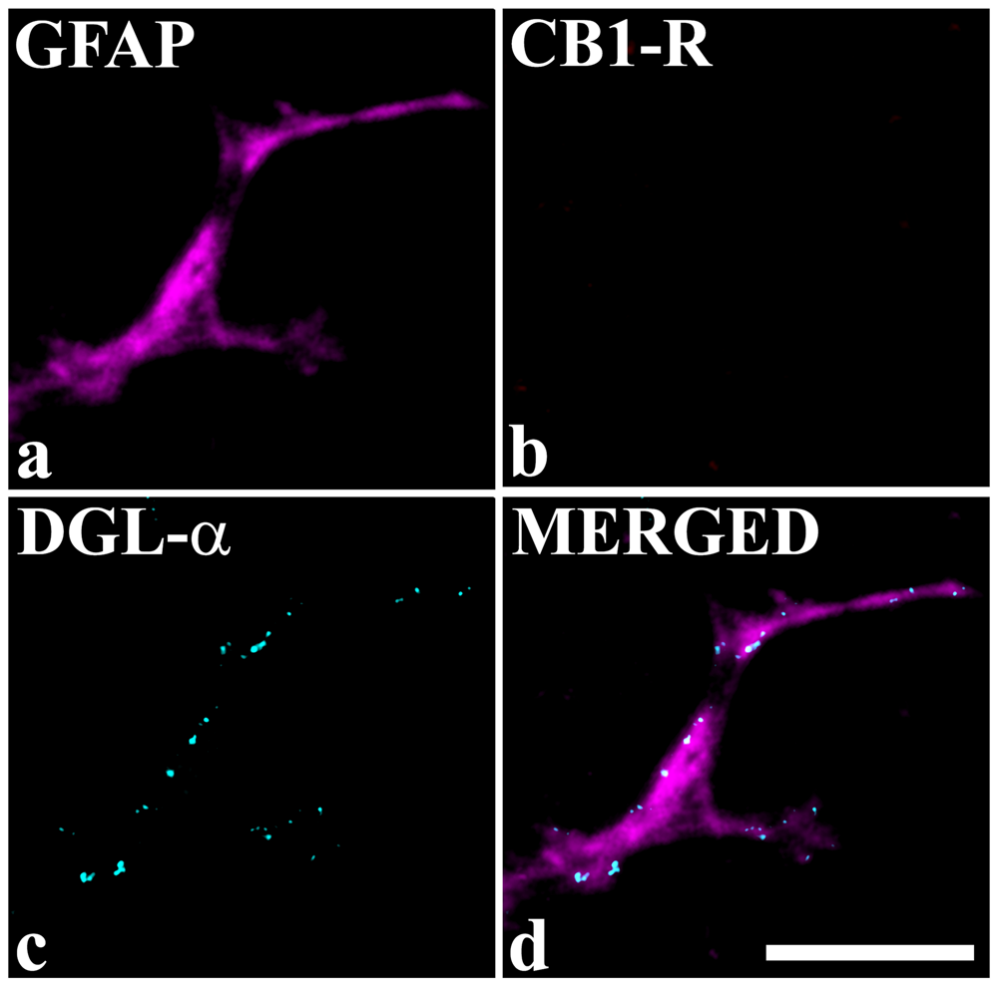
Astrocytes cultured from CB_1_-R knock out mice do not express CB_1_-Rs. Micrographs of a single 1 µm thick laser scanning confocal optical section illustrating the co-localization between immunolabeling for GFAP (a marker for astrocytes, magenta,; (**a,d**) and DGLα (cyan; **c,d**), and the lack of immunostaining for CB_1_-R (**b**) in the process of an astrocyte cultured from CB_1_-R knock out mouse. The merged image on **d** shows that puncta immunoreactive for DGLα are located within the confines of the glial process stained for GFAP. Scale bar: 10 µm.

In cultures obtained from spinal cords of CB_1_-R knock out mice, GFAP-IR astrocytes showed a strong immunolabeling for DGLα, as expected (Fig. 4c,d), but they were all negative for CB_1_-Rs (Fig. 4b,d). In these cultures, we investigated 122 GFAP-IR astrocytes from which 20.4 ± 2.7% displayed immunostained puncta for DGLα.

Thus, fulfilling our expectations, in a substantial proportion of cultured rat and mouse spinal astrocytes, similarly to astrocytes in the superficial spinal dorsal horn, immunostaining for CB_1_-R and DGLα showed a high degree of spatial co-localization.

### Activation of CB_1_-Rs evokes Ca^2+^ transients in cultured spinal astrocytes

The activation of CB_1_-Rs increased [Ca^2+^]_i_ in astrocytes within the superficial spinal dorsal horn. Therefore, we next verified whether the activation of CB_1_-Rs on cultured astrocytes also results in a transient increase in [Ca^2+^]_i_.

**Figure 5:**
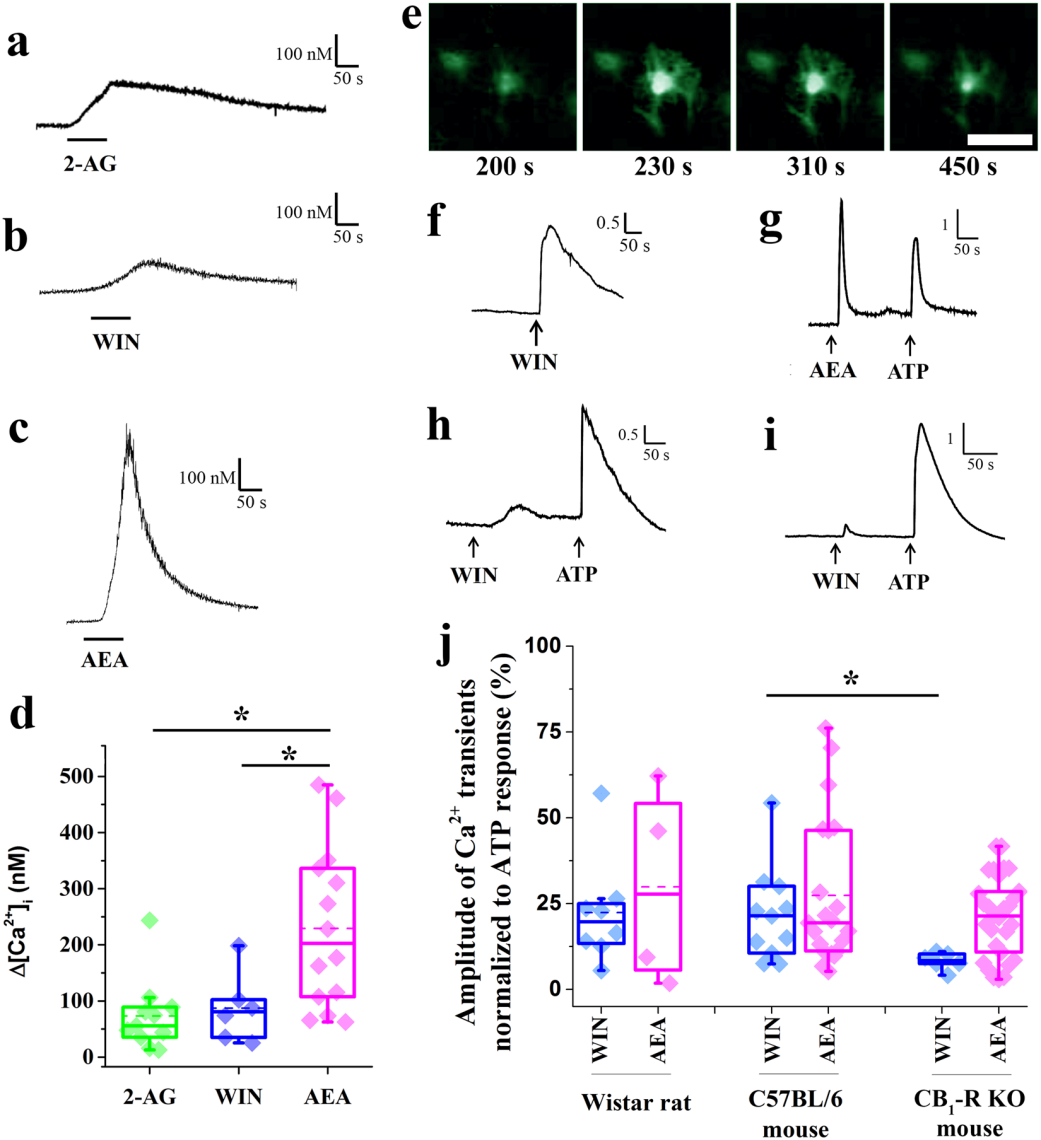
Activation of CB_1_-Rs evokes Ca^2+^ transients in cell bodies of cultured spinal astrocytes. (**a–c**) Graphical representation of changes in [Ca^2+^]_i_ recorded in rat cultured astrocytes following the application of 10 µM 2-AG (**a**), 10 µM WIN (**b**), and10 µM anandamide (AEA) (**c**). (**d**) Box plot illustrating the amplitudes of calcium transients of rat cultured astrocytes in response to the application of 10 µM 2-AG, 10 µM WIN, and 10 µM AEA. Asterisks indicate significant differences between the amplitudes of calcium transients evoked by 2-AG, WIN and AEA (p = 0.0007, 2-AG vs AEA; p = 0.0354 WIN vs AEA). **e**: Micrographs showing the fluorescent intensity of a Fluo-8-AM-loaded cultured rat spinal astrocyte at different time points after the application of 10 µM WIN. Scale bar: 50 µm. (**f–i**) Graphical representations of the time courses of calcium transients evoked by the application of 10 µM WIN (**f**), 10 µM anandamide and 180 µM ATP (**g**) in cultured rat spinal astrocytes, and 10 µM WIN and 180 µM ATP in astrocytes cultured from wild type (**h**) and CB_1_-R knock out (**i**) mice. (**j**) Box plot showing the amplitudes of the calcium transients normalized to ATP responses in the responding cells cultured from rats, wild type mice, and CB_1_–R knock out mice. Dashed and continuous lines within the boxes represent the mean and median values of the data sets, respectively. Asterisks indicate significant difference between the amplitudes of calcium transients evoked by WIN in wild type and CB_1_-R knock out mice (p = 0.0118).

First, we carried out whole cell calcium measurements on cultured rat astrocytes treated with CB_1_-R agonists. Specifically, cells were loaded with the calcium sensitive dye Fura-2-AM, and a CB_1_-R agonist (anandamide, 2-AG or WIN) was applied directly to the recorded cells. Fura-2 was alternately excited with a monochromatic light of 340 and 380 nm, and the emitted fluorescent signal was continuously monitored. The resting [Ca^2+^]_i_ calculated as the average of [Ca^2+^]_i_ of all recorded cells before CB_1_-R agonists treatment, was 72 ± 3 nM. Astrocytes responded to the application of CB_1_-R agonists with slow calcium transients, of which the full width at half maximum was 135.6 ± 29.0, 151.8 ± 19.5 and 74.1 ± 6.5 seconds, in response to the application of 2-AG, WIN and anandamide, respectively (Fig. 5a–c). Although all applied agonists evoked responses in some of the cultured astrocytes, different agonists induced changes in [Ca^2+^]_i_ to very different extents: anandamide was the most efficacious activator of CB_1_-R, increasing the [Ca^2+^]_i_ by 237 ± 38 nM (Fig. 5c,d), whereas 2-AG and WIN raised the [Ca^2+^]i by only 63 ± 10 nM and 94 ± 25 nM, respectively (Fig. 5a,b,d; p = 0.4808, 2-AG vs WIN; p = 0.0354, anadamide vs WIN; and p = 0.0007 anandamide vs 2-AG,).

To verify that the increase in [Ca^2+^]_i_ in the cultured rat astrocytes was the result of CB_1_-R activation, and to compare the efficacy of CB_1_-R agonists on rat and mouse astrocytes, we carried out another set of experiments on astrocytes cultured from rat, wild type and CB_1_-R knock out mice. We did not test the effect of 2-AG in these experiments, because the effect of 2-AG on [Ca^2+^]_i_ turned out to be very similar to that induced by WIN in the earlier experiment, and WIN is thermodynamically stable whereas 2-AG is prone to isomerization over time^30^. Cells were loaded with the calcium indicator Fluo-8-AM and the fluorescence intensity within the cell body of individual astrocytes was then measured with a confocal laser scanning microscope, both at rest and after the application of the CB_1_-R agonists WIN and anandamide. The experiments were run for 350–450 seconds and images were taken at one frame per second during the recording. After the effect of the applied CB_1_-R agonist completely vanished, ATP was added to the cultures and its effect on [Ca^2+^]_i_ was also recorded. Only those cells that exhibited Ca^2+^ transients in response to ATP were considered alive and included in the analysis.

In rat astrocyte cultures, 4.3 ± 0.5% and 4.9 ± 1.2% of all living cells responded with calcium transients to WIN and anandamide, respectively (Fig. 5e–g). The amplitudes of the evoked calcium signals were 22.3 ± 5.5% (for WIN) and 29.9 ± 14.48% (for anandamide) of those induced by ATP (Fig. 5f,g,j, Table 1). In cultures obtained from wild type mice, 8.3 ± 0.7% and 18.3 ± 1.2% of living astrocytes responded with a normalized F_n_ value of 21.61 ± 4.1% and 27.4 ± 4.7% to WIN and anandamide, respectively (Fig. 5h,j, Table 1). The cannabinoid-evoked responses in rat and mouse astrocytes were very similar (p = 0.20 for WIN in rat vs WIN in mouse and p = 0,72 for anandamide in rat vs anadamide in mouse; Fig. 5j, Table 1).

**Table 1:** Numbers of cultures and cultured astrocytes in which calcium transients evoked by the application of WIN, anandamide and ATP were measured.

animal type	drug	n (cultures)	n (cells responding to ATP)	n (cells responding to drug)	proportion of ATP sensitive cells responding to drug	amplitude of Ca^2+^ transient normalized to ATP response (%)
Wistar rat	WIN	15	233	10	4.3 ± 0.5	22.33 ± 5.52
	AEA	10	122	6	4.9 ± 1.2	29.87 ± 14.48
C57BL/6 mouse	WIN	6	157	13	8.3 ± 0.7	21.61 ± 4.12
	AEA	8	202	37	18.3 ± 1.2	27.37 ± 4.68
CB_1_-R KO mouse	WIN	9	400	10	2.5 ± 0.2	8.28 ± 1.01
	AEA	12	505	27	5.3 ± 0.3	19.28 ± 2.14

The proportion of astrocytes responding to the application of WIN, anandamide and ATP, as well as the amplitudes of the calcium transients recorded from the responding cells, normalized to ATP responses, are also presented. n: number, AEA: anandamide.

In cultures obtained from CB_1_-Rs knock out mice, we observed a remarkable reduction both in the proportion of ATP sensitive cells responding to the applied drugs and in the amplitude of the evoked transients (Fig. 5i, Table 1). Only 2.5 ± 0.2% and 5.3 ± 0.3% of the living cells responded to the application of WIN and anandamide with a normalized F_n_ value of 8.28 ± 1.01% and 19.28 ± 2.14%, respectively (Fig. 5i,j, Table 1). Comparing these values to those obtained in wild type mice, the amplitudes of calcium transients evoked by WIN were significantly lower in astrocytes cultured from CB_1_-R knock out mice (p = 0.0118; wild type vs CB_1_-R knock out mice; Fig. 5j, Table 1). We also observed a slight reduction in the case of anandamide application, but it was not significant (p = 0.6654).

Although nearly 30% of cultured astrocytes were positively stained for CB_1_-Rs (presented in the previous section), much lower proportions of them responded to the application of CB_1_-R agonists WIN and anandamide with Ca^2+^ transients. These seemingly contradictory findings may be explained by the fact that we measured [Ca^2+^]_i_ within the cell bodies of astrocytes, whereas the evoked calcium transients, like the CB_1_-Rs may be primarily confined to astrocytic processes. Reinforcing this notion, Di Castro *et al*.^27^ reported intense synaptic activity-driven local Ca^2+^ activity in short segments of processes of mature astrocytes in the dentate gyrus.

### CB_1_-R agonists evoke Ca^2+^ transients primarily in micro-domains of processes and only occasionally in cell bodies of cultured spinal astrocytes

**Figure 6:**
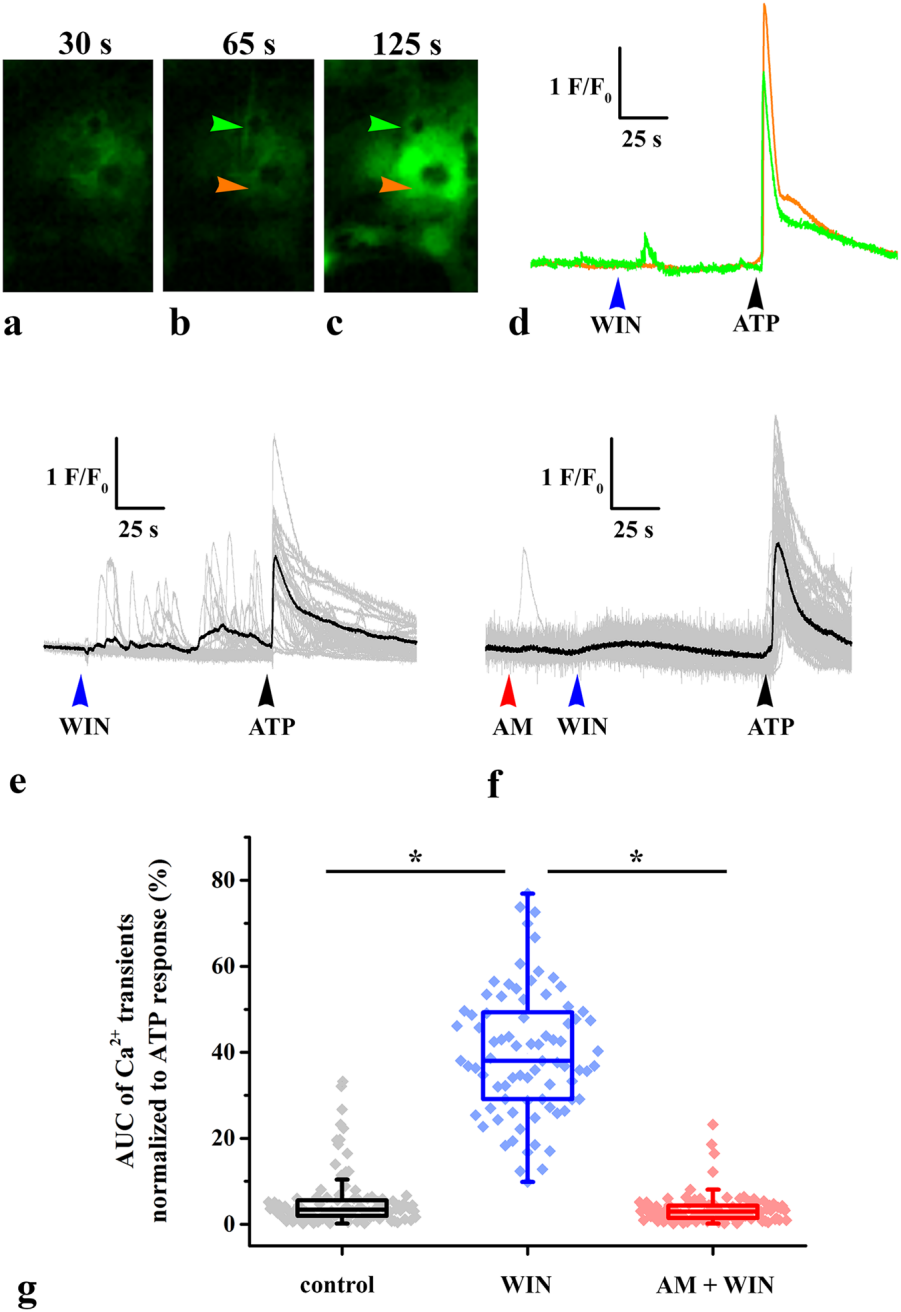
Activation of CB_1_-Rs evokes Ca^2+^ transients in the processes of cultured mouse spinal astrocytes. (**a**–**c**) Micrographs showing the fluorescence intensity of a Fluo-8 loaded astrocytic process (green arrowhead) and astrocytic cell body (orange arrowhead) before (**a**) and after the application of 10 µM WIN (**b**) and 180 µM ATP (**c**). (**d**) Graphical representation of changes in fluorescence intensities of the process (green trace) and the cell body (orange trace) which are labeled with green and orange arrowheads on (**a**–**c**) respectively, before and after the application of 10 µM WIN and 180 µM ATP. Note that both the process and cell body responded to ATP, whereas only the process showed activity following the administration of WIN. (**e**–**f**) Individual (gray) and average (black) Ca^2+^ transients in cultured astrocyte processes following the application of 10 µM WIN alone (**e**) and after a pretreatment with 5 µM AM251. In both cases, cell viability was tested by the application of 180 µM ATP. Only those processes that exhibited Ca^2+^ transients in response to the application of ATP were considered alive and included in the consecutive statistical analysis. (**g**) Box plot illustrating the area under the curve (AUC) values (normalized to ATP response) of Ca^2+^ transients recorded from processes of cultured astrocytes under control conditions, as well as following the administration of WIN without and with a preincubation of the cells with AM251. Asterisks indicate significant differences between AUC values of calcium transients recorded in the different experimental conditions (p = 8.81 × 10^−31^, control vs WIN; p < 0.001, the Mann-Whitney test provided the value of 0, WIN vs AM + WIN).

To test whether the application of CB_1_-R agonists evoke local Ca^2+^ transients in spinal astrocytes similar to that reported by Di Castro *et al*.^27^, we carried out calcium imaging on cultured mouse spinal astrocytes with a spinning disc confocal system equipped with a high sensitivity sCMOS camera. After loading the cells with the calcium sensitive dye Fura-8-AM and recording the spontaneous activity of astrocytes, we applied WIN to the bath solution. Similar to the previous observations, WIN application occasionally evoked calcium transients in cell bodies. We recorded calcium transients in 12 (8.9%) of the 134 investigated cell bodies. However, we also observed for the first time in spinal astrocytes that substantial elevations in [Ca^2+^]_i_ also occurred in short segments of glial process (Fig. 6a–d). These local Ca^2+^ signals appeared in large numbers (26.6 ± 6.4 active segments in a 700 × 350 µm large field of view; Fig. 6b,c).

To check the viability of astrocytes, two minutes after the application of WIN, ATP was added to the cultures and its effect on [Ca^2+^]_i_ was also recorded (Fig. 6c–e). WIN evoked activity was evaluated only for those microdomains of astrocytic processes that exhibited Ca^2+^ transients in response to the application of ATP. Finally, we evaluated WIN evoked calcium transients in 80 segments of astrocytic processes (Fig. 6e). To compare the calcium events recorded before and after the application of WIN, we calculated the AUC for the control period and for the drug application period, then normalized the values to the ATP responses. We found that the average AUC of the calcium signals during the control period was 5.4 ± 0.6% of the ATP response, which increased to 39.7 ± 1.7% (p = 8.81 × 10^–31^) after the application of WIN (Fig. 6g).

In some cases, prior to the WIN application, cells were pretreated with the inverse CB_1_-R agonist AM251, which completely abolished the effects of WIN (Fig. 6f,g). Following AM251 pretreatment, WIN application had no effect on the basal activity of the cells; the average AUC value calculated from recordings of 109 segments of astrocytic processes was 3.4 ± 0.3% of the ATP response.

Thus, we verified for the first time, that the activation of CB_1_-R evokes local Ca^2+^ transients in short segments of processes of spinal astrocytes.

### Cultured spinal astrocytes release 2-AG after activation of their CB_1_-Rs

We next explored whether the elevated [Ca^2+^]_i_ in astrocytic processes was sufficient to activate DGLα, resulting in the production and release of 2-AG by cultured spinal astrocytes.

Cultured spinal astrocytes isolated from wild-type mice were stimulated by adding WIN into the culture medium. To evoke a CB_1_-R independent general increase in [Ca^2+^]_i_, other cultures were treated with the calcium ionophore A23187 which elevates [Ca^2+^]_i_ by increasing the calcium permeability of cell membranes. In addition, two other sets of experiments were run, in which astrocytes were pre-incubated with the inverse CB_1_-R agonist AM251 (to block CB_1_-R activation) or with the selective chelator of intracellular Ca^2+^ BAPTA-1-AM (to prevent calcium transients evoked by CB_1_-R activation) before the administration of WIN. After the stimulations, the cells were scraped and the mixture of the cells and the medium was collected. Lipids were extracted from the cell suspensions and 2-AG, anandamide, and two other anandamide-like N‐acylethanolamides, oleoylethanolamide (OEA) and palmitoylethanolamide (PEA) were pre-purified and quantified by isotope dilution-liquid chromatography-atmospheric pressure chemical ionization-mass spectrometry (LC-APCI-MS).

**Figure 7:**
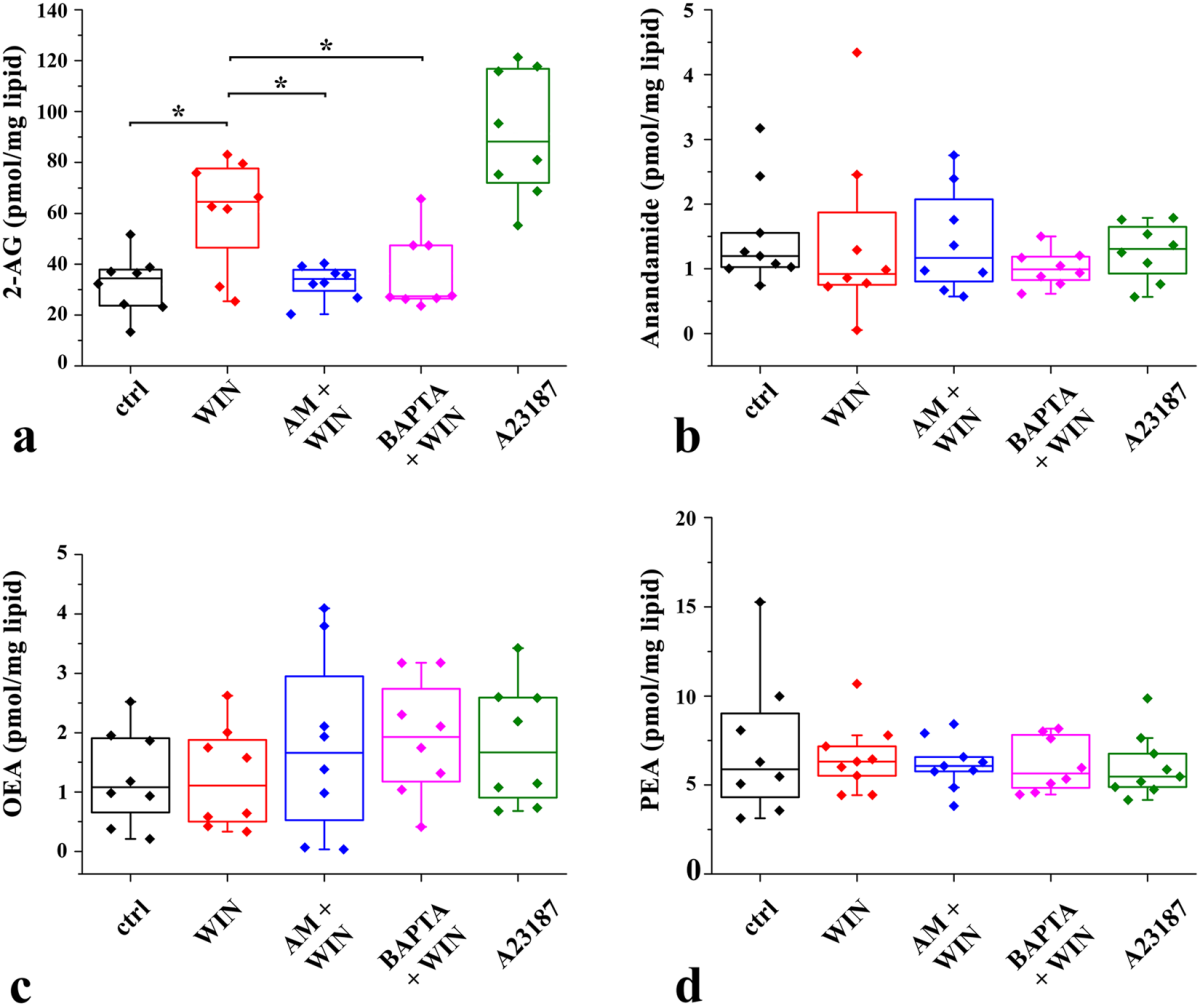
In response to the activation of their CB_1_-Rs, cultured spinal astrocytes produce and release 2-AG, but not anandamide, oleoylethanolamide (OEA) or palmitoylethanolamide (PEA). Box-plots showing the quantities of 2-AG (**a**), anandamide (**b**), OEA (**c**) and PEA (**d**) in pmol/mg measured with LC-APCI-MS in cultured wild-type mouse spinal astrocytes. Quantities of endocannabinoids were measured in control conditions and following the application of 10 µM WIN or the Ca^2+^ ionophore A23187 (5 µM). The effects of 5 µM AM251 and 5 µM BAPTA-1-AM pretreatment on WIN-evoked 2-AG production are also illustrated. Asterisks indicate significant differences between the quantities of 2-AG measured under different experimental conditions (p = 0.023, ctrl vs WIN; p = 0.040, WIN vs AM + WIN; p = 0.041, WIN vs BAPTA + WIN).

The LC-APCI-MS data showed that the cultured mouse spinal astrocytes produced 32.1 ± 4.1 pmol/mg 2-AG under basal conditions (Fig. 7a), in accordance with earlier publications^20^. The application of WIN significantly increased the level of 2-AG to 60.7 ± 7.6 pmol/mg (p = 0.023) (Fig. 7a). The WIN evoked response can be regarded as substantial, because even the calcium ionophore A23187 increased the resting level of 2-AG to only 91.3 ± 8.8 pmol/mg (Fig. 7a). Although A23187 had a greater impact on 2-AG production than WIN, the effects of the two compounds were not significantly different (p = 0.052). On the other hand, we found only 32.9 ± 2.3 pmol/mg (p = 0.96 vs basal) and 36.4 ± 5.3 pmol/mg (p = 0.71 vs basal) 2-AG in samples pretreated with AM251 or BAPTA-1 AM, respectively, before the application of WIN (Fig. 7a). These data demonstrate that the CB_1_-R inverse agonist AM251 and the calcium chelator BAPTA-1-AM completely prevented WIN from inducing 2-AG synthesis. These results indicate that WIN acts on CB_1_-Rs and subsequently increases [Ca^2+^]_i_, in micro-domains of astrocytic processes. The increase in [Ca^2+^]_i_, activate DGLα resulting in an increased synthesis of 2-AG.

In addition to 2-AG, the cultured spinal astrocytes also produced 1.2 ± 0.1 pmol/mg anandamide, 1.2 ± 1.3 pmol/mg OEA and 6.0 ± 5.4 pmol/mg PEA under basal conditions (Fig. 7b–d). In contrast to 2-AG, however, the administration of either WIN or A23187 did not alter the levels of these compounds (Fig. 7b–d), indicating that their production was unaffected by activation of CB_1_-Rs and subsequent elevation of [Ca^2+^]_I_.

## Discussion

Here we investigated whether spinal astrocytes can release 2-AG due to the activation of their CB_1_-Rs. First in the literature, we showed that rat spinal astrocytes both in spinal cord slices and in culture co-express CB_1_-Rs and DGLα in close proximity to each other. We also demonstrated that activation of CB_1_-Rs evokes a substantial elevation in [Ca^2+^]_i_ in micro-domains of processes of cultured astrocytes. Finally, we revealed that Ca^2+^ transients evoked by the activation of CB_1_-Rs induce the production of the endocannabinoid 2-AG in cultured spinal astrocytes. The results provide direct evidence for the existence of a cannabinoid induced cannabinoid release mechanism in spinal astrocytes. This new discovery unifies previously separated and seemingly unrelated ideas^16,17,22–24^, and indicates that, in addition to neuron-neuron communication, endocannabinoids also play a major role in neuron-astrocytes-neuron bidirectional signaling. In addition, our results also imply that the bidirectional communication between neurons and astrocytes can be mediated by both anandamide and 2-AG from neurons to astrocytes, and mostly, if not exclusively by 2-AG from astrocytes to neurons.

### Ca^2+^ transients evoked by CB_1_-R activation

Here, we provided evidence that activation of CB_1_-Rs on spinal astrocytes, as in the hippocampus^16,17^ and in other brain areas^31–37^, results in a substantial increase in [Ca^2+^]_i_. Importantly, although we applied endocannabinoids anandamide and 2-AG at concentrations that can activate receptors other than CB_1_-R (e.g. GPR 55, TRPV1, CB_2_), the control experiments verified that our results were not considerably biased by these off-target effects. Indeed, the proportion of astrocytes responding to WIN or anandamide in cultures obtained from CB_1_-R knock out animals was less than one-third of those in culture from wild type mice, suggesting that the applied agonists exerted their effect mostly through CB_1_-Rs. Some astrocytes isolated from CB_1_-R knock out animals, however, did respond to WIN and anandamide. Although these calcium transients may represent spontaneous cellular activity coincided with the application of the drugs, we cannot exclude the possibility that some spinal astrocytes may express low levels of additional cannabinoid-sensitive receptors such as GPR55, TRPV1 or CB_2_. The investigation of the roles of these receptors was beyond the scope of our present study, but one has to be aware that the expression of these receptors on astrocytes would substantially diversify the cannabinoid-mediated signaling pathways between neurons and astrocytes.

It is important to note that only a fraction of spinal astrocytes were sensitive to cannabinoids. Although it was previously shown that approximately 50% of astrocytic profiles in the superficial spinal dorsal horn of rats display immunoreactivity for CB_1_-Rs^19^, here we recovered CB_1_-Rs on only approximately 30% of cultured spinal astrocytes. Because we isolated astrocytes for culturing from the whole spinal cord, the difference in the previous and present data may indicate that CB_1_-Rs occur more abundant in astrocytes within the superficial spinal dorsal horn than in other areas of the spinal cord. The distribution of immunoreactivity for CB_1_-R within the spinal gray matter reinforces this notion^19^. Thus, previous and our present results suggest that endocannabinoid mediated neuron-astrocyte interactions may play a more substantial role in spinal pain processing than in other functions of the spinal cord, given the known importance of the superficial spinal cord in pain processing.

### 2-AG production evoked by CB_1_-R activation

In the hippocampus, endocannabinoids released by neurons activate CB_1_-Rs on astrocytes, which leads to the mobilization of Ca^2+^ from internal stores^16,17^. A specific consequence of increased [Ca^2+^]_i_ in astrocytes is the release of gliotransmitters, such as glutamate, D-serine and ATP^2,12,38,39^. In addition, an increase in [Ca^2+^]_i_ evoked by ATP or endothelin^20,22,23^ can also activate the PLC-DGLα cascade, resulting in the production of 2-AG^20–22,28^. However, 2-AG production evoked by CB_1_-Rs activation has never been demonstrated. Filling up this gap in our knowledge, here we reported for the first time in the literature that the activation of CB_1_-Rs evokes an increase in [Ca^2+^]_i_ which induces 2-AG mobilization in spinal astrocytes. The astrocytic 2-AG-mediated signaling mechanism may represent an important form of astrocyte-to-neuron communication, because, depending on the spatial distribution and actual activation pattern of excitatory and inhibitory synapses expressing CB_1_-Rs, the release of 2-AG from astrocytes can either depress or enhance the net activity of local neural circuits within a given spatial compartment^39,40^. Hence, astrocytes as targets and sources of endocannabinoids may substantially contribute to endocannabinoid-dependent plasticity.

### Endocannabinoid-mediated bidirectional communication between neurons and astrocytes in the superficial spinal dorsal horn

The endocannabinoid signaling apparatus is activated by neural activities^16^. In the superficial spinal dorsal horn, in particular, neural activity is primarily initiated by glutamatergic nociceptive primary afferents, which activate spinal neurons. In the case of repetitive stimulation, postsynaptic neurons can release 2-AG from perisynaptic membrane compartments^26,41^. The released 2-AG binds to presynaptic CB_1_-Rs resulting in a transient depression of synaptic activity. A fraction of the 2-AG, however, can diffuse out from the site of release to a distance of 20 µm^17^ and activate CB_1_-Rs on adjacent neurons and astrocytes^16,20,22,23,42^. The activation of CB_1_-Rs on astrocytes then leads to PLC-dependent Ca^2+^ mobilization in astrocytic processes^16,24,43–45^. Considering the possibility that the incoming primary afferents may evoke complex, spatially and temporally distributed patterns of synaptic activity in the spinal dorsal horn, several astrocytic micro-compartments may respond with Ca^2+^ transients reflecting the original pattern of synaptic activity. The increased [Ca^2+^]_i_ in the activated compartments may activate PLC and DGLα, resulting in further release of 2-AG from astrocytes. This may act locally but may also diffuse out and act on neural CB_1_-Rs, thereby affecting the functional properties of spinal neurons remote from the termination field of the originally activated nociceptive primary afferents^46^. Thus, because of the involvement of astrocytes, the primary afferent-evoked original activity, initially limited to a relatively small area, may ultimately influence the functional properties of excitatory and inhibitory synapses in a much wider field. This mechanism may play a substantial role in the tonic tuning of neural excitability, the spike timing precision of neurons, and eventually the modulation of complex network functions^15^, such as pain processing in neural assemblies of the superficial spinal dorsal horn. Although the various mechanisms of actions underlying the endocannabinoid modulation of spinal nociceptive functions remain to be fully elucidated, CB_1_-R-mediated neuron-astrocyte-neuron bidirectional communication should be considered when interpreting the effects of (endo)cannabinoids on spinal pain processing.

## Methods

### Animals

Experiments were carried out on adult and new born (7–9 day old) rats (Wistar-Kyoto, 250–300 g, Gödöllő, Hungary), C57BL6 wild type and CB_1_-R knock out mice (kind gift of Andreas Zimmer, Bonn, Germany). All animal study protocols were approved by the Animal Care and Protection Committee at the University of Debrecen, and were in accordance with the European Community Council Directives.

To validate experimental findings obtained from wild type animals it was essential to conduct identical parallel experiments on wild type and CB_1_-R knock out animals. For this reason, the use of mice was crucial for the present project. On the other hand, however, several earlier studies addressing the problem of spinal endocannabinoid mediated neuron-glia communication were performed in rats. Because we would have liked to compare our present results also to the earlier rat studies, we conducted the most critical experiments also in rats.

### Preparation of tissue sections

For immunocytochemical detection of CB_1_-Rs and DGLα, the animals were deeply anesthetized with sodium pentobarbital (50 mg/kg, i.p.) and transcardially perfused with Tyrode’s solution (oxygenated with a mixture of 95% O_2_, 5% CO_2_), followed by a fixative containing 4% paraformaldehyde dissolved in 0.1 M phosphate buffer (PB, pH 7.4). After transcardial fixation, the L3-L5 segments of the spinal cord were removed, post-fixed in their original fixative for 1–4 hours, and immersed into 10% and 20% sucrose dissolved in 0.1 M PB until they sank. To enhance reagent penetration, the removed spinal cord was freeze-thawed in liquid nitrogen. Transverse sections (50 µm thick) were cut on a vibratome and washed thoroughly in 0.1 M PB.

### Cell culture of spinal astrocytes

Primary cell cultures of spinal astrocytes were generated from rats, wild-type mice and CB_1_-R knockout mice (all 7–9 days old). After decapitation, the spinal cord was removed and placed into ice-cold dissecting buffer (136 mM NaCl, 5.2 mM KCl, 0.64 mM Na_2_HPO_4_, 0.22 mM KH_2_PO_4_, 16.6 mM glucose, 22 mM sucrose, 10 mM HEPES supplemented with 0.06 U/ml penicillin and 0.06 U/ml streptomycin), in which the meninges were carefully removed. The spinal cords were incubated in a solution containing 0.025 g/ml bovine trypsin (catalog no.: T4799, Sigma, St Louis, USA) for 30 minutes at 37 °C, then transferred into a tissue culture medium (minimum essential medium, catalog no.: 21090-022, Life Technologies, New York, USA) supplemented with 10% fetal bovine serum (catalog no.: F2442, Sigma, St. Louis, USA) for 5 minutes at room temperature. Tissue pieces were gently suspended by a Pasteur pipette. The suspension was filtered on a nylon mesh and centrifuged for 10 minutes at 800 rpm. Isolated cells with a density of 6 × 10^5^/ml were placed onto tissue culture dishes coated with 0.3 mg/ml poly-L-lysine or onto coverslips that were put into wells of tissue culture plates. Non-adherent cells were removed on the second day of culture. Adherent cells were cultured for 10–12 days in a CO_2_ incubator (CO_2_ concentration: 5%, humidity: 95%) at 37 °C. The tissue culture medium was changed every second day. The purity of the cultures was regularly controlled with staining cell nuclei with DAPI and immunocytochemical detection of markers specific for astrocytes (GFAP), microglial cells (CD11b) and neurons (NeuN). Only those cultures were used for the experiments in which the purity of astrocytes, the proportion of GFAP-IR cells, was more than 80% (a generally accepted purity for primary cultures). Some cells (2–8%) showed positive staining for NeuN, and CD11b immunoreactive cells were occasionally also recovered. Most of the non-GFAP-IR cells, however, turned out to be negative for both NeuN and CD11b.

### Immunohistochemistry

Triple immunostaining protocols were performed to study the GFAP, CD11b and NeuN immunoreactivity of cultured cells as well as the co-localization of GFAP, CB_1_-R and DGLα immunoreactivity in astrocytes within the spinal cord and in cell cultures obtained from rats as well as from wild type and CB_1_-R knock out mice.

#### Triple immunostaining for GFAP, CD11b and NeuN

Astrocyte cultures generated on coverslips from rats, wild type and CB_1_-R knock out mice were first incubated with a mixture of antibodies that contained mouse anti-GFAP (diluted 1:1000, catalog no.: MAB3402, Millipore, Temecula, California, USA), rabbit anti-CD11b (1:500, catalog no.: T3102, Bachem, Bubendorf, Switzerland) and guinea pig anti-NeuN (1:500, catalog no.: 266004, Synaptic System, Göttingen, Germany) for overnight at 4 °C. Cultures were then transferred into a mixture of goat anti-mouse IgG conjugated with Alexa Fluor 488 (1:1000, catalog no.: A-11001, Invitrogen, Eugene, Oregon, USA), goat anti rabbit IgG conjugated with Alexa Flour 555 (1:100 A-21428, Invitrogen, Eugene, Oregon, USA) and goat-and guinea pig IgG conjugated with Alexa Fluor 647 (1:1000, catalog no.: A-21450, Invitrogen, Eugene, Oregon, USA) for 4 hours at room temperature. Before the antibody treatments the cultures were kept in 10% normal goat serum (catalog no.: S-1000, Vector Labs., Burlingame, California, USA) for 50 minutes. Antibodies were diluted in 10 mM TPBS (pH 7.4) containing 1% normal goat serum (catalog no.: S-1000, Vector Laboratories, Burlingame, California, USA). The immunostained cultures were covered with VectaShield-DAPI (catalog no.: H-1200, Vector Laboratories, Burlingame, California, USA).

#### Triple immunostaining for GFAP, CB_1_-R and DGLα

Tissue sections and astrocyte cultures obtained from rat, wild type and CB_1_-R knock out mice spinal cords were first incubated with a mixture of antibodies that contained mouse anti-GFAP (1:1000, catalog no.: MAB3402, Millipore, Temecula, California, USA), rabbit anti-CB_1_-R (1:1000, catalog no.: 10006590, Cayman Chemical, Ann Arbor, Michigan, USA) and goat anti-DGLα (1:500, catalog no.: Af1080, Frontier Institute, Hokkaido, Japan). The sections and cultures were then transferred for overnight incubation in a mixture of donkey anti-mouse IgG conjugated with Alexa Fluor 488 (1:1000, catalog no.: A-21202, Invitrogen, Eugene, Oregon, USA), donkey anti-rabbit IgG conjugated with Alexa Fluor 647 (1:1000, catalog no.: A-31573, Invitrogen, Eugene, Oregon, USA) and donkey anti-goat IgG conjugated with Alexa Fluor 555 (1:1000, catalog no.: A-21432, Invitrogen, Eugene, Oregon, USA) secondary antibodies. Before the antibody incubations, the sections were kept in 10% normal donkey serum (catalog no.: ab7475, Abcam, Cambridge, UK) for 50 minutes. Antibodies were diluted in 10 mM TPBS (pH 7.4) containing 1% normal donkey serum (catalog no.: ab7475, Abcam, Cambridge, UK). Sections were mounted on glass slides and covered with VectaShield-DAPI (catalog no.: H-1200, Vector Laboratories., Burlingame, California, USA).

### Immunohistochemical controls

The specificity of the primary antibody against CB_1_-R and DGLα has been extensively characterized previously in various immunostaining protocols and Western blots and also in knock out animals^19,26,47,48^.

### Confocal microscopy and analysis

Single and short series of 1-µm-thick optical sections (15 optical sections with an overlap of 0.5 µm) were scanned from the spinal dorsal horn and cell cultures, using an Olympus FV1000 confocal microscope with a 60x oil-immersion lens (NA: 1.4). The confocal settings (laser power, confocal aperture and gain) were identical for all methods, and care was taken to ensure that no pixels corresponding to puncta immunostained for GFAP, CB_1_-R and DGL-α were saturated. The scanned images were processed by Adobe Photoshop CS5 software.

The co-localization of CB_1_-R and DGLα within the confines of GFAP immunoreactive astrocytic profiles was quantitatively analyzed in triple-stained sections and cell cultures.

For spinal cord sections, a 10 × 10 standard square grid in which the edge-length of the unit square was 4 µm was placed onto the regions of single confocal images corresponding to laminae I-II of the superficial spinal dorsal horn. The following anatomical features were used to ensure proper placement of the grid: (a) the border between the dorsal column and the dorsal horn, which was easily identified on the basis of the intensity of immunostaining. (b) the border between laminae II and III, which was approximated on the basis of previous observations^49,50^. It has been demonstrated in ultrastructural studies that there are almost no myelinated axons in lamina II, whereas they are abundant in lamina III. Thus, the border between laminae II and III can be defined quite precisely in ultrastructural studies, and the thickness of laminae I-II can be measured. Therefore, immunoreactivities and co-localizations were investigated in the most superficial 150 µm thick zone of the dorsal horn that has previously been identified as the layer of the gray matter corresponding to laminae I and II in segments L3-L5 of the spinal dorsal horn.

Profiles that showed immunoreactivity for GFAP and was located on the edges of the standard grid were counted in the medial and lateral compartments of laminae I and II. The selected profiles were then examined to determine whether they were also immunoreactive for CB_1_-R or DGLα. CB_1_-R and DGLα immunolabeled puncta were always smaller than the area immunostained for GFAP. In addition, since the CB_1_-R and DGLα antibodies used in the present study were raised against the intracellular domain of CB_1_-R or DGL-α, immunolabeled puncta were expected to be located within the confines of the area immunostained for the markers. Thus, to define the co-localization values we counted only those CB_1_-R or DGLα immunolabeled puncta that were located within the confines of the areas immunostained for GFAP. The co-localization was analyzed in three animals. The quantitative measurement was carried out in three sections that were randomly selected from each animal. Thus, the calculations for quantitative figures were based on the investigation of nine sections.

For cell cultures, the quantitative measurement was carried out on three independent cultures. We took five images of single optical sections from each triple stained culture in such a way that the images did not show any overlap with each other. Thus, the calculation of quantitative figures was based on the investigation of 15 images (regions of interest, ROI). We identified all GFAP immunoreactive astrocytes within the 15 ROIs. The processes of the selected astrocytes were then examined to determine whether they were also immunoreactive for CB_1_-R or DGLα.

The distances between CB_1_-R-IR and the closest DGLα-IR puncta were measured on short series of 1 µm thick optical sections both in the spinal cord and cell cultures. The sampling procedure was identical to that described above for the single optical sections. The z-stack images were imported into Imaris software (Imaris version 7.7.2., Bitplane) and GFAP immunostained astrocytic processes were segmented out manually with the Imaris Surface module (http://www.bitplane.com/imaris/imaris;nif-0000-00314). The segmented processes were then exported as surface objects within Imaris to run a distance transformation on the outside as well as inside of the surface objects to select the CB_1_-R-IR and DGLα-IR spots associated with the surface of the segmented process. The distance transformation was done with the “distance transformation” XTension of the Imaris XT module (http://bitplane.com/imaris/imarisxt;nif-0000-00314). The CB_1_-R-IR and DGLα-IR puncta were segmented with Imaris Spots retraction feature. The Spot objects on the surface of processes were then selected by using a filter on the resulting channel from the distance transformation. Finally, the distances between the CB_1_-R-IR puncta and the closest DGLα-IR spots were measured.

### Calcium imaging and loose patch extracellular recording in spinal cord tissue slices

#### Slice preparation

Experiments were carried out on 10–15 days old C57BL6 mice. The animals were decapitated, the lumbar segments of the spinal cords were removed and transferred into ice-cold low-sodium artificial cerebrospinal fluid containing (in mM) NaCl, 25; sucrose, 130; glycerol, 60; NaHCO_3_, 26; NaH_2_PO_4_, 1.25; glucose, 10; KCl, 2.5; ascorbic acid, 0.5; CaCl_2_, 1; MgCl_2_ 2 (all from Sigma-Aldrich, St. Louis, MO, USA). Cross-sectional slices with a thickness of 200 μm were cut with a vibratome, and the slices were transferred into an incubation chamber containing artificial cerebrospinal fluid (aCSF) which contained (in mM): NaCl, 125; KCl, 2.5; NaHCO_3_, 26; glucose, 10; NaH_2_PO_4_, 1.25; CaCl_2_, 2; MgCl_2_, 1; myo-inositol, 3; ascorbic acid, 0.5; sodium pyruvate, 2 (all from Sigma-Aldrich,St. Louis, MO, USA). The aCSF was continuously bubbled with a mixture of 95% O_2_ and 5% CO_2_ at 33 °C. Electrical recordings and calcium imaging were carried out in recording chambers, in which oxygenated aCSF was continuously perfused over the slices at room temperature.

#### Calcium imaging

For fluorescent calcium imaging, spinal cord slices were incubated in a 40 μM solution of the acetoxymethylester form of the fluorescent dye Oregon Green 488 BAPTA-1 (Invitrogen-Molecular Probes, Carlsbad, CA, USA) dissolved in oxygenated aCSF at 33 °C for 20–30 minutes. After recording the calcium transients of cells at rest in aCSF, TTX (1 μM) was applied to the bath. To test the effect of CB_1_-R activation on cells that showed slow calcium transients even in the presence of TTX, the perfusion solution was changed to aCSF containing 1 μM WIN (catalog no.: 1038, Tocris Bioscience, Bristol, UK) and 1 μM TTX. The investigated cells were illuminated with a light beam the wavelength of which was set at 488 nm by the monochromator of the Polychrome V light source (Till Photonics GmbH, Gräfelfing, Germany). Images were collected at a frame rate of 10 Hz with a Zeiss Axioskop microscope (Carl Zeiss AG, Oberkochen, Germany) equipped with a CCD camera (SensiCam, PCO AG, Kelheim, Germany), with 20x and 40x water immersion objectives. The fluorescent filter set contained a dichroic mirror (Omega XF2031 505DRLPXR; Omega Drive, Brattleboro, VT, USA), an emission filter (LP 515, Till Photonics) and a 1.4-megapixel CCD camera (SensiCam, PCO AG, Kelheim, Germany). Following data acquisition, the fluorescent transients of recorded cells were further processed and analyzed with the Image J software package. The fluorescent bleaching that occurred during the recording was corrected to a stable baseline value, and the fluorescent traces were converted to a ∆F/F_0_% form.

#### Extracellular loose patch recording

For extracellular loose-patch recording of neuronal activities, micropipettes (resistance: 5–6 MΩ) filled with aCSF were moved towards cells until seal resistances of ≥50 MΩ were achieved. The electrical activity of the cells was recorded in voltage-clamp mode using an Axopatch 200 A amplifier (Molecular Devices, Union City, CA, USA). Data acquisition was performed at a sampling rate of 10-kHz with a ‘gap-free’ protocol using the Clampex 10.2 software (Molecular Devices, Union City, CA, USA). Off-line filtering and data analysis of the loose-patch current recordings were performed using the Clampfit 10.2 program (Molecular Devices).

#### Intracellular biocytin labeling

Some cells showing slow calcium transients were intracellularly labeled with biocytin. Micropipettes (resistance: 5–6 MΩ) were filled with a solution containing (in mM): K-gluconate, 120; NaCl, 5; HEPES [4-(2-hydroxyethyl)-1- piperazineethanesulfonic acid], 10; EGTA, 2; CaCl2, 0.1; Mg-ATP, 5; Na3-GTP, 0.3; Na2-phosphocreatinine, 10; biocytin, 8. In whole-cell patch clamp recording mode, the cells were filled with the internal solution of the micropipettes. The slices containing the labeled cells were fixed in 4% paraformaldehyde dissolved in 0.1 M PB (pH 7.4) for overnight at 4 °C. The slices were incubated with streptavidin-conjugated Alexa 488 (diluted 1:300; Molecular Probes Inc., Eugene, OR, USA) for 90 min and then covered with VectaShield-DAPI (catalog no.: H-1200, Vector Labs., Burlingame, California, USA).

### Calcium imaging of cell cultures

#### Calcium imaging with a confocal laser scanning microscope

Cultured cells were incubated in a solution of 4 µM Fluo-8-AM (catalog no.: ABD-21080, AAT Bioquest, Sunnyvale, CA) for 30 minutes at 37 °C. The cultures were then transferred into artificial cerebrospinal fluid (aCSF) containing (in mM): NaCl, 135; KCl, 3; glucose, 10; CaCl_2_, 2; MgCl_2_, 1; HEPES, 10 (pH 7.2; 305 mOsm/kg; all from Sigma, St Louis, USA). Calcium imaging was performed at room temperature under an LSM 510 Meta confocal laser scanning microscope (Zeiss, Oberkochen, Germany) equipped with an argon ion laser. After recording the spontaneous activity of the cultured cells, anandamide (catalog no.: 1339, AEA; Tocris Bioscience, Bristol, UK) or WIN (catalog no.: 1038, Tocris Bioscience, Bristol, UK) was added to the aCSF bath solution in a final concentration of 10 μM. Then, ATP (catalog no.: A7699, Sigma, St Louis, USA) was added to the bath at a final concentration of 180 µM to check the viability of the recorded cells.

Series of images were recorded using a 10x objective (NA: 0.3) with a scanning speed of one frame/second. The scanned images were analyzed by Image J. The fluorescence intensity values of all recorded cells at each recorded time points were measured. F_n_ values for each recorded data were calculated according to the equation of F_n_ = F/F_o_, where F is the measured mean fluorescence intensity and F_o_ is the mean background fluorescent intensity. F_n_ values were depicted according to the time scale of the records. The amplitudes of Ca^2+^ transients evoked by the application of anandamide and WIN were normalized to the responses evoked by the application of ATP according to the following equation: (response to drug/response to ATP) ×100.

#### Calcium imaging with a spinning disc confocal system

Cultured cells were incubated in a solution of 1 µM Fluo-8-AM in the presence of 0.01% pluronic acid at room temperature for 30 minutes. The cultures were then transferred into artificial cerebrospinal fluid (aCSF) containing (in mM): NaCl, 135; KCl, 3; glucose, 10; CaCl_2_, 2; MgCl_2_, 1; HEPES, 10 (pH 7.2; 305 mosm/kg; all from Sigma, St Louis, USA). Ca^2+^ imaging was carried out with an Andor Zyla 5.5 sCMOS camera attached to a differential spinning disk (DSD2, Andor Technology) built on an Olympus IX-81 inverted microscope. Using a 20x objective (NA:0.45), images of 540 × 306 pixels (corresponding to a field of view of 235 × 130 µm, which was populated with 50 to 100 cells) were acquired at five frames per second with Andor iQ3 software. Cells filled with Fluo-8-AM were excited at 488 nm, and emitted fluorescence was collected at 520 nm. Acquisition parameters (illumination intensity, exposure time, readout time, frame rate) were identical for all experiments. After recording the spontaneous activity of the astrocytes, 10 µM WIN was applied to the bath solution. In some cases, cells were pretreated with the inverse CB_1_-R agonist AM251 in a final concentration of 5 µM prior to the application of WIN. As a final treatment, 180 µM ATP was administered to the bath solution to verify the viability of the cells. Changes in fluorescence intensities were measured over astrocyte processes by drawing freehand region of interests around the processes that showed either spontaneous or drug-induced activity. Changes in [Ca^2+^]_i_ were estimated as changes of the fluorescence signal over baseline (dF/F_o_, where F_o_ was the average initial fluorescence). A region of interest was considered to respond to the application of a compound if dF/F_o_ was at least three times the standard deviation of the baseline for at least five consecutive images. Experimental data were analyzed with Microsoft Excel 2013 (Microsoft), and area under the curve (AUC) calculations were performed with Origin Pro 8.0 (OriginLab, Northampton, MA). For each traces, the ratio of AUC values induced by WIN and ATP was calculated.

### Whole cell calcium measurements

Cultured cells were incubated in a solution of 10 μM Fura-2 AM (catalog no.: F-1201, Life Technologies, Budapest, Hungary) for 1 hour at 37 °C. The cultures were transferred into aCSF for 30 minutes at room temperature and placed onto the stage of an inverted fluorescent microscope (Diaphot; Nikon, Tokyo, Japan). The cultured cells within the recording chamber were continuously perfused with oxygenated aCSF while the CB_1_-R agonists anandamide (catalog no.: 1339, Tocris Bioscience, Bristol, UK), 2-AG (catalog no.: 62160, Cayman Chemical, Ann Arbor, USA) or WIN (catalog no.: 1038, Tocris Bioscience, Bristol, UK) in 10 μM concentration were directly applied onto the cells through a capillary tube (Perfusion Pencil™; AutoMate Scientific, San Francisco, CA, USA) with the aid of a local perfusion system (Valve Bank™ 8 version 2.0, AutoMate Scientific). The agonist was carefully washed out, then 180 µM ATP (catalog no.: A7699, Sigma, St Louis, USA) was puffed onto the recorded cells to check their viability.

The investigated cells were illuminated with a light beam for which the wavelength was switched between 340 and 380 nm by a dual-wavelength monochromator (Deltascan, Photon Technology International, New Brunswick, NJ, USA), while the emission was monitored at 510 nm using a photomultiplier. [Ca^2+^]_i_ values (R) were calculated from the ratio of the recorded fluorescence intensities (R = F_340_/F_380_). The resting and the evoked [Ca^2+^]_i_ values were calculated and plotted against time.

### Harvesting cells and culture medium following the activation of cell cultures with CB_1_-R agonists

Primary cell cultures of wild-type mouse spinal astrocytes were generated as described above. On days 10–12 in culture, the culture medium was replaced with 500 µL Hank’s Balanced Salt Solution (HBSS). Cells were maintained in HBSS for 30 minutes and then treated with 10 µM WIN or the ionophore A23187 (5 µM) for 2.5 minutes. In two other sets of experiments, cells were treated with WIN following a preincubation of the astrocytes with 5 µM AM251 or 5 µM BAPTA-1-AM. The stimulation was stopped by adding 500 µL ice-cold methanol. Cells were scraped, and an additional 500 µL of methanol was pipetted into the wells. Thus, we collected a sample solution of 1500 µL from each well, containing both the cells and supernatant. For the quantification of 2-AG under basal conditions, cells were maintained in 500 µL HBSS for 32.5 min. The treatment with WIN and ATP was omitted, but the application of methanol was identical to the sample preparation steps mentioned above. All samples were stored at −80 °C for liquid chromatography – mass spectrometry.

### Liquid chromatography – mass spectrometry

Lipids were extracted from cell suspensions and 2-AG, anandamide, palmitoylethanolamide (PEA) and oleoylethanolamide (OEA) pre-purified and quantified using LC-APCI-MS as described previously^51^. First, cells were Dounce-homogenized and extracted with chloroform/methanol/Tris-HCl 50 mM pH 7.5 (2:1:1, v/v) containing internal deuterated standards (5 pmol) for anandamide, 2-AG, PEA and OEA quantification by isotope dilution ([^2^H]_8_ AEA, [^2^H]_5_ 2-AG, [^2^H]_4_ PEA, [^2^H]_4_ OEA (Cayman Chemicals, MI, USA). The lipid-containing organic phase was dried down, weighed and pre-purified by open-bed chromatography on silica gel. Fractions were obtained by eluting the column with 99:1, 90:10 and 50:50 (v/v) chloroform/methanol. The 90:10 fraction was used for anandamide, 2-AG, PEA and OEA quantification by LC-APCI-MS, as previously described and using selected ion monitoring at M + 1 values for the four compounds and their deuterated homologues, as described previously^52^.

### Statistics

Before the statistical analysis, outliers were identified (Q1-3IQR or Q3 + 3IQR, where Q is quartile and IQR is interquartile range) in each data set and were excluded from further analysis. Sample sizes were not predetermined; however, post hoc power tests were performed to verify that they are adequate for quantifying the experimental data. Box-and-whisker plots were generated, and all statistical analyses were performed using Origin Pro 8.0 software. Data sets were compared with the Wilcoxon signed rank test (Fig. 2i,j) or the two-tailed nonparametric Mann-Whitney U test (Figs 5d,j and 6g, 7a). Equal variances between data sets were assumed. Differences were considered significant when *p* < 0.05. In the text, data sets are presented as mean ± SEM.

### Data availability

All relevant data are available from the authors.
