# Author Correction: CB_1_ receptor activation induces intracellular Ca^2+^ mobilization and 2-arachidonoylglycerol release in rodent spinal cord astrocytes

**DOI:** 10.1038/s41598-018-30573-9

**Published:** 2018-09-07

**Authors:** Zoltán Hegyi, Tamás Oláh, Áron Kőszeghy, Fabiana Piscitelli, Krisztina Holló, Balázs Pál, László Csernoch, Vincenzo Di Marzo, Miklós Antal

**Affiliations:** 1 Department of Anatomy, Histology and Embryology, Faculty of Medicine, University of Debrecen, 4032 Debrecen, Hungary; 2 Department of Physiology, Faculty of Medicine, University of Debrecen, 4032 Debrecen, Hungary; 3 Endocannabinoid Research Group, Institute of Biomolecular Chemistry, Consiglio Nazionale delle Ricerche, 80078 Pozzuoli, Naples Italy; 4 MTA-DE Neuroscience Research Group, University of Debrecen, 4032 Debrecen, Hungary; 5 Present Address: Department of Cognitive Neurobiology, Center for Brain Research, Medical University of Vienna, 1090 Vienna, Austria

Correction to: *Scientific Reports* 10.1038/s41598-018-28763-6, published online 12 July 2018

The original version of this Article contained a typographical error in the spelling of the author Fabiana Piscitelli, which was incorrectly given as Fabiana Pisticelli. This has now been corrected in the PDF and HTML versions of the Article.

